# Vitamin D Deficiency as an Important Biomarker for the Increased Risk of Coronavirus (COVID-19) in People From Black and Asian Ethnic Minority Groups

**DOI:** 10.3389/fpubh.2020.613462

**Published:** 2021-01-22

**Authors:** Shahina Pardhan, Lee Smith, Raju P. Sapkota

**Affiliations:** ^1^Faculty of Health, Education, Medicine and Social Care, Vision and Eye Research Institute (VERI), School of Medicine, Anglia Ruskin University, Cambridge, United Kingdom; ^2^Faculty of Science and Engineering, The Cambridge Centre for Sport and Exercise Sciences, Anglia Ruskin University, Cambridge, United Kingdom

**Keywords:** COVID-19, vitamin D deficiency, risk factor, Black and Asian communities, coronavirus infection 2019

## Introduction

Ever since the new 2019 coronavirus disease (COVID-19) was first identified in Wuhan, Hubei province of China its spread has become a global pandemic affecting almost every country worldwide. World Health Organization (WHO) has confirmed more than 71 million cases of COVID-19 and over 1,620,000 deaths globally (until 16 December 2020) (1), and the numbers are increasing rapidly.

There is growing evidence to suggest that people from Black (mostly African) and Asian (mostly South Asians/South East Asians) ethnic groups are disproportionately affected by COVID-19, leading to poorer outcomes (higher mortality and morbidity) compared to White British or Americans (2–4). Public Health England (in August 2020) reported that Black people are 2–3 times more likely to be infected with COVID-19 compared to White people after adjusting for age (5). A study from 260 hospitals across England, Scotland and Wales found that people from Black and South Asian backgrounds were, 36 and 28% respectively, more likely to be admitted for critical care, after adjusting for age, gender and deprivation of area lived (6). Data from the intensive care units showed that people from Black and Asian ethnic groups accounted for more than 25% of all COVID-19 admissions (until end of July 2020) (7), despite comprising only about 11% of the total population of the UK. COVID-19 related deaths within Black and Asian ethnic groups working in the health care settings in the UK was even higher (63%) (8, 9).

In Chicago, USA, more than 50% of the total COVID-19 cases and nearly 70% of COVID-19 related deaths were reported in Black people, although they comprised only about 30% of Chicago's total population (10). Centers for Disease Control and Prevention reported that the rate of coronavirus (COVID-19) infection was 2.6 times higher, hospitalization 4.7 times higher and deaths 2.1 times higher in Black/African Americans compared to White (Non-Hispanic) Americans (11). In Asians, the rate of coronavirus infection reported was 1.1 times higher and hospitalization 1.3 times higher compared to White (Non-Hispanic) Americans.

Various reasons have been offered to explain why people from Black and Asian ethnic minority groups are more at risk of coronavirus infection and mortality. These include socio-demographic factors, underlying heath issues, overcrowded households, living in deprived areas, difficulty in health care access due to language barriers, unhealthy lifestyles, and performing “higher-risk” frontline healthcare or essential work (9). However, research suggests that even after adjusting for age, gender, lifestyles, socio-economic factors, language barriers, self-reported health/disability conditions, people from Black and Asian ethnic groups were still more likely to be infected and die from COVID-19 than White people (12, 13). In the UK, data show that COVID-19 related deaths were 1.9 times higher in Black people and 1.6–1.8 times higher in Asians compared to White people, after adjusting or age, socio-economic characteristics and self-reported health/disability measures (13).

In exploring these health and social determinants of inequality in ethnic minorities, differences in other factors such as low levels of Vitamin D have not been addressed adequately. Vitamin D deficiency poses a potential risk factor for COVID-19. Vitamin D deficiency is identified as a risk factor in older age, diabetes, obesity, and hypertension (14, 15) which are significantly associated with COVID-19 (16). Recent studies showed negative correlations between mean vitamin D levels and COVID-19 cases across different European countries including Spain, Italy, and Switzerland and in the US (17, 18). Although it is appreciated that correlations do not suggest causality, these findings cannot be discounted. There are number of limitations and methodological differences in these studies. Ile et al. (17) is an ecological study reporting only crude associations, and findings may be limited by the fact that the number of positive cases are directly affected by the proportion of COVID-19 tests performed, which may vary between countries. In Kaufman et al. (18) study, vitamin D data were obtained within the preceding 12 months and hence may not all be up-to-date. Also, it is likely that findings from the study may not be representative of the general population as participants who took part belonged to certain priority groups such as those who had symptoms, had come in contact with people who had tested positive or who belonged to the “high-risk” categories for COVID-19 infections.

Significant ethnic variations in the gene GC that encodes Vitamin D binding protein (DBP) (protein that circulates Vitamin D/metabolites in the blood) have been reported. Black people and Asians are more likely to carry the GC1F variant of this (GC) gene, which has been associated with low DBP levels, and lower synthesis and metabolism of Vitamin D (19). On the other hand, white people are more likely to carry the GC1S variant in whom higher DBP levels are generally observed (20).

It is known that darker skin in Black people and Asians can lead to a lower concentration of vitamin D in the blood as the increased melanin in their skin reduces the absorption of sunlight needed to produce vitamin D (21, 22). It is likely that lower exposure to sunlight, for example, with cultural attire, may also contribute to reduced vitamin D concentration as would more time spent indoors during lockdown.

Serum 25-hydroxyvitamin D level of <50 nmol/L (20 ng/mL) is classified as Vitamin D deficiency in adults (23). In Europe, people from the dark-skinned ethnic background were found to be more at risk of vitamin D deficiency compared to white counterparts (22). Vitamin D deficiency has also been reported in infants, adults and pregnant women of Asian families living in the UK (24–26).

Vitamin D supplementation could reduce the risk of influenza and COVID-19 infections and mortality (27) by reducing the viral replication rates and expression of pro-inflammatory cytokines which injure the lining of the lungs, leading to pneumonia, thereby providing a protection against COVID-19 (28). Therefore, it is important vitamin D deficiency should not be overlooked as an important risk factor for COVID-19 in Black and Asian ethnic groups in whom vitamin D deficiency is more prevalent.

It is important to acknowledge that the effect of vitamin D deficiency on COVID-19 and its outcomes can be confounded by obesity, that in itself poses additional risk for viral infections, their progression and recovery. Vitamin D deficiency has been shown to be higher in obese individuals. A systematic review and meta-analysis, published in 2015, shows a 35% higher prevalence of vitamin D deficiency in obese individuals (29). In addition, obesity has been shown as an additional risk for other viral infections such as from H1N1 and influenza A with delayed recovery time (30, 31).

The affected immune system in COVID-19 is thought to play an important role in obesity-induced adipose tissue inflammation and metabolic dysfunctions such as diabetes, hypertension, and cardiovascular disease (32). These underlying conditions, known to be significant risk factors for COVID-19 complications, are more prevalent in people from Asian countries including India, Pakistan, and Bangladesh (33). The role played by body mass index (BMI) in COVID-19 was shown by data from China (34), in which 88% of people who did not survive had a higher BMI (>25 kg/m^2^) compared to 19% who survived, suggesting obesity poses a significant additional risk for COVID-19 infection and its progression.

Vitamin D has been stipulated as a risk factor in other virus infections in people from ethnic minorities. A cross-sectional study of vitamin D levels in 200 HIV-infected patients in south-central US (Houston, Texas) found that nearly two-thirds (64%) of patients were vitamin D deficient, and that African-American (in whom HIV infection was more prevalent) were over three times (odds ratio = 3.53) more likely to have vitamin D deficiency compared to White Americans (35). In the UK, data from 1077 HIV patients, showed that 73.5% of patients had vitamin D deficiency, with Black patients 3 times more likely to be deficient (36). In Spain, a hospital-based study showed that HIV patients from non-Caucasian background were 3.18 times more likely to have vitamin D deficiency than from Caucasian background (37).

Ethnic differences in vitamin D levels are also reported in patients infected with Hepatitis B virus (HBV) and Hepatitis C Virus (HCV). A study on African American and White Americans infected with HCV found that vitamin D deficiency was significantly greater in African Americans (44%) compared to White Americans (15%) (38). A recent systematic review and meta-analysis (39) of seven studies reported significantly reduced vitamin D levels in patients infected with HBV than in healthy controls, with the highest reduction in Indian patients (40).

One study by Hastie et al. (41), reported lack of evidence on the potential link between vitamin D levels and the risk of COVID-19 infection in people from Black and Asian ethnic groups. The baseline data on vitamin D levels, ethnicity, underlying health conditions, socioeconomic status, etc. of participants enrolled in the UK Biobank (between 2006 and 2010) were examined against those participants who tested positive for COVID-19 in 2020. However, a number of limitations in the study need to be taken into account. The data on Vitamin D and health status were obtained a decade ago and these were then examined for participants who tested positive in 2020. It is likely that these might have changed significantly over the course of 10 years. In addition, their analysis is based on only 32 Black people, 19 south Asians and 13 people from other ethnicities who were hospitalized with COVID-19 over a month (between 16 March and 17 April 2020, short time frame). We argue that this is not representative of the general population and more research on larger numbers is needed.

## Conclusion

Evidence suggests vitamin D deficiency plays an important role in the high rate of infection and mortality of COVID-19 in Black and Asian ethnic minority groups, but more research is needed to confirm this. This should be a priority for future research including large clinical trials in order to better understand the vulnerability of these ethnic groups and ascertain the effectiveness of using vitamin D supplements to reduce the risk of COVID-19 infection, severity and mortality. A number of trials have led the way and examined the role of vitamin D or its analogs/metabolites [e.g., calcitriol, calcifediol, 1,25(OH)2D3] in preventing and treating COVID-19 (42–44). Findings suggest that calcitriol exhibits significant potent activity against the coronavirus infection (42), and a high dose of Calcifediol/25-hydroxyvitamin D reduces the need for intensive care treatment (43). Another trial showed that patients who received vitamin D had improved clinical recovery as evidenced by shorter lengths of hospital stay, lower oxygen requirements, and reduced inflammatory markers (44). Whilst promising, there are a number of limitations to these findings including small sample sizes and selected cohorts such as people who were hospitalized. It is also not clear whether vitamin D analogs/metabolites would benefit people at an earlier stage of the disease. The possible role of obesity was not considered (43) and the effects on ethnicity has not been examined in detail on a larger sample of people (44). While these results are encouraging, larger trials are needed to draw firmer conclusions. Obviously, other health and social determinants influencing the high risk of COVID-19 facing Black people and Asians should not be overlooked.

## Author Contributions

SP and RS drafted the manuscript. SP, RS, and LS revised the manuscript. All authors contributed to the article and approved the submitted version.

## Conflict of Interest

The authors declare that the research was conducted in the absence of any commercial or financial relationships that could be construed as a potential conflict of interest.

## References

[1] World Health Organisation Coronavirus Disease (COVID-19) Dashboard.

[2] Kirby T. Evidence mounts on the disproportionate effect of COVID-19 on ethnic minorities. Lancet Respir Med. (2020) 8:547–8. 10.1016/S2213-2600(20)30228-9 32401711 PMC7211498

[3] Moorthy A Dubey S Samanta A Adebajo A Aggarwal A Jain A. COVID-19 and ethnicity: Spotlight on the global rheumatology issues in developing and developed countries. Int J Rheum Dis. (2020) 23:849–52. 10.1111/1756-185X.13883 32473047 PMC7300781

[4] Vepa A Bae JP Ahmed F Pareek M Khunti K. COVID-19 and ethnicity: a novel pathophysiological role for inflammation. Diabetes Metab Syndr. (2020) 14:1043–51. 10.1016/j.dsx.2020.06.056 32640416 PMC7326443

[5] Public Health England Disparities in the Risks and Outcomes of COVID-19. London (2020).

[6] Harrison EM Docherty AB Barr B Iain B Carson G Drake TM Ethnicity and Outcomes from COVID-19: The ISARIC CCP-UK Prospective Observational Cohort Study of Hospitalised Patients (05/31/20). Available online at: https://ssrn.com/abstract=3618215

[7] Intensive Care National Audit and Research Centre (ICNARC) Report on COVID-19 in Critical Care. London (2020).

[8] Rimmer A. Covid-19: Two thirds of healthcare workers who have died were from ethnic minorities. BMJ. (2020) 369:m1621. 10.1136/bmj.m1621 32327412

[9] Cook T Kursumovic E Lennane S Exclusive: deaths of NHS staff from covid-19 analysed. Health Service J. (2020) 22:04.

[10] Yancy CW. COVID-19 and African Americans. JAMA. (2020) 323:1891–2. 10.1001/jama.2020.6548 32293639

[11] Centers for Disease Control and Prevention COVID-19 Hospitalization and Death by Race/Ethnicity. Atlanta, GA (2020).

[12] Niedzwiedz CL O'Donnell CA Jani BD Demou E Ho FK Celis-Morales C. Ethnic and socioeconomic differences in SARS-CoV-2 infection: prospective cohort study using UK Biobank. BMC Med. (2020) 18:160. 10.1186/s12916-020-01640-8 32466757 PMC7255908

[13] Office of National Statistics UK Coronavirus (COVID-19) Related Deaths by Ethnic Group. London (2020).

[14] Pearce SH Cheetham TD. Diagnosis and management of vitamin D deficiency. BMJ. (2010) 340:b5664. 10.1136/bmj.b5664 20064851

[15] Laird E Rhodes J Kenny RA. Vitamin D and inflammation: potential implications for severity of Covid-19. Ir Med J. (2020) 113:81. 32603576

[16] Wu Z McGoogan JM. Characteristics of and important lessons from the Coronavirus Disease 2019. (COVID-19) Outbreak in China: Summary of a report of 72 314 cases from the Chinese Center for Disease Control and Prevention. JAMA. (2020). 323:1239–42. 10.1001/jama.2020.2648 32091533

[17] Ilie PC Stefanescu S Smith L. The role of vitamin D in the prevention of coronavirus disease 2019 infection and mortality. Aging Clin Exp Res. (2020) 32:1195–8. 10.1007/s40520-020-01570-8 32377965 PMC7202265

[18] Kaufman HW Niles JK Kroll MH Bi C Holick MF. SARS-CoV-2 positivity rates associated with circulating 25-hydroxyvitamin D levels. PLoS ONE. (2020) 15:e0239252. 10.1371/journal.pone.0239252 32941512 PMC7498100

[19] Nizamutdinov I Popov Y Ilinsky V Rakitko A Allele frequency distribution of SNPs associated with levels of Vitamin D-binding protein and 25-hydroxyvitamin D. bioRxiv. (2019) 2019:564229 10.1101/564229

[20] Powe CE Evans MK Wenger J. Vitamin D-binding protein and vitamin D status of black Americans and white Americans. N Engl J Med. (2013) 369:1991–2000. 10.1056/NEJMoa1306357 24256378 PMC4030388

[21] Nair R Maseeh A. Vitamin D: The sunshine vitamin. J Pharmacol Pharmacother. (2012) 3:118–26. 10.4103/0976-500X.95506 22629085 PMC3356951

[22] Cashman KD Dowling KG Škrabáková Z Gonzalez-Gross M Valtueña J De Henauw S. Vitamin D deficiency in Europe: pandemic? Am J Clin Nutr. (2016) 103:1033–44. 10.3945/ajcn.115.120873 26864360 PMC5527850

[23] Holick MF. Vitamin D deficiency. N Engl J Med. (2007) 357:266–81. 10.1056/NEJMra070553 17634462

[24] Shaw NJ Pal BR. Vitamin D deficiency in UK Asian families: activating a new concern. Arch Dis Child. (2002) 86:147–9. 10.1136/adc.86.3.147 11861227 PMC1719105

[25] Akhtar S. Vitamin D status in South Asian populations - risks and opportunities. Crit Rev Food Sci Nutr. (2016) 56:1925–40. 10.1080/10408398.2013.807419 25746099

[26] Guo J Lovegrove JA Givens DI. A narrative review of the role of foods as dietary sources of vitamin D of ethnic minority populations with darker skin: The underestimated challenge. Nutrients. (2019) 11:81. 10.3390/nu11010081 30609828 PMC6356726

[27] Grant WB Lahore H McDonnell SL Baggerly CA French CB Aliano JL Evidence that vitamin D supplementation could reduce risk of influenza and COVID-19 infections and deaths. Nutrients. (2020) 12:988 10.3390/nu12040988 PMC7231123 32252338

[28] Rhodes JM Subramanian S Laird E Kenny RA. Editorial: low population mortality from COVID-19 in countries south of latitude 35 degrees North supports vitamin D as a factor determining severity. Aliment Pharmacol Ther. (2020) 51:1434–7. 10.1111/apt.15777 32311755 PMC7264531

[29] Pereira-Santos M Costa PR Assis AM Santos CA Santos DB. Obesity and vitamin D deficiency: a systematic review and meta-analysis. Obes Rev. (2015) 16:341–9. 10.1111/obr.12239 25688659

[30] Milner JJ Rebeles J Dhungana S Stewart DA Sumner SC Meyers MH. Obesity increases mortality and modulates the lung metabolome during pandemic H1N1 influenza virus infection in mice. J Immunol. (2015) 194:4846–59. 10.4049/jimmunol.1402295 25862817 PMC4417391

[31] Maier HE Lopez R Sanchez N Ng S Gresh L Ojeda S. Obesity increases the duration of influenza A virus shedding in adults. J Infect Dis. (2018) 218:1378–82. 10.1093/infdis/jiy370 30085119 PMC6151083

[32] Kassir R. Risk of COVID-19 for patients with obesity. Obes Rev. (2020) 21:e13034. 10.1111/obr.13034 32281287 PMC7235532

[33] Eapen D Kalra GL Merchant N Arora A Khan BV. Metabolic syndrome and cardiovascular disease in South Asians. Vasc Health Risk Manag. (2009) 5:731–3. 10.2147/VHRM.S5172 19756165 PMC2742703

[34] Peng YD Meng K Guan HQ Leng L Zhu RR Wang BY. Clinical characteristics and outcomes of 112 cardiovascular disease patients infected by 2019-nCoV]. Zhonghua Xin Xue Guan Bing Za Zhi. (2020) 48:450–5. 10.3760/cma.j.cn112148-20200220-00105 32120458

[35] Crutchley RD Gathe J Jr Mayberry C Trieu A Abughosh S Garey KW. Risk factors for vitamin D deficiency in HIV-infected patients in the south central United States. AIDS Res Hum Retroviruses. (2012) 28:454–9. 10.1089/aid.2011.0025 21878055

[36] Welz T Childs K Ibrahim F Poulton M Taylor CB Moniz CF. Efavirenz is associated with severe vitamin D deficiency and increased alkaline phosphatase. AIDS. (2010) 24:1923–8. 10.1097/QAD.0b013e32833c3281 20588161

[37] Lerma E Molas ME Montero MM Guelar A González A Villar J. Prevalence and factors associated with vitamin D deficiency and hyperparathyroidism in HIV-infected patients treated in Barcelona. ISRN AIDS. (2012) 2012:485307. 10.5402/2012/485307 24052874 PMC3767361

[38] White DL Tavakoli-Tabasi S Kanwal F Ramsey DJ Hashmi A Kuzniarek J. The association between serological and dietary vitamin D levels and hepatitis C-related liver disease risk differs in African American and white males. Aliment Pharmacol Ther. (2013) 38:28–37. 10.1111/apt.12341 23710689 PMC3742078

[39] Hu YC Wang WW Jiang WY Li CQ Guo JC Xun YH. Low vitamin D levels are associated with high viral loads in patients with chronic hepatitis B: a systematic review and meta-analysis. BMC Gastroenterol. (2019) 19:84. 10.1186/s12876-019-1004-2 31185932 PMC6558894

[40] Sajith KG Kapoor N Shetty S Goel A Zachariah U Eapen CE. Bone health and impact of tenofovir treatment in men with hepatitis-B related chronic liver disease. J Clin Exp Hepatol. (2018) 8:23–27. 10.1016/j.jceh.2017.05.009 29743793 PMC5938523

[41] Hastie CE Mackay DF Ho F Celis-Morales CA Katikireddi SV Niedzwiedz CL Vitamin D concentrations and COVID-19 infection in UK Biobank. Diabetes Metab Syndr. (2020) 14:561–5. 10.1016/j.dsx.2020.04.050 32413819 PMC7204679

[42] Mok C Ng Y Ahidjo B Lee RCH Loe MWC Liu J Calcitriol, the active form of vitamin D, is a promising candidate for COVID-19 prophylaxis. bioRxiv [Preprint]. (2020). 10.1101/2020.06.21.162396

[43] Entrenas Castillo M Entrenas Costa LM Vaquero Barrios JM Alcalá Díaz JF López Miranda J Bouillon R. Effect of calcifediol treatment and best available therapy versus best available therapy on intensive care unit admission and mortality among patients hospitalized for COVID-19: A pilot randomized clinical study. J Steroid Biochem Mol Biol. (2020) 203:105751. 10.1016/j.jsbmb.2020.105751 32871238 PMC7456194

[44] Ohaegbulam K Swalih M Patel P Smith M Perrin R. Vitamin D supplementation in COVID-19 patients: A clinical case series. Am J Ther. (2020) 27:e485–e90. 10.1097/MJT.0000000000001222 32804682 PMC7473790

